# Comparative analysis of toxicity in patients with anal cancer undergoing definitive simultaneous integrated boost (SIB) or sequential integrated boost (SeqB) radiotherapy

**DOI:** 10.1007/s00384-023-04411-y

**Published:** 2023-05-12

**Authors:** Margherita Rotondi, Giuseppe Facondo, Stefano Mossa, Gianluca Vullo, Ilaria Angelicone, Maurizio Valeriani, Mattia Falchetto Osti

**Affiliations:** Department of Medicine and Surgery and Translational Medicine, Radiotherapy Oncology, Sapienza University of Rome, St. Andrea Hospital, 00189 Rome, Italy

**Keywords:** Radiotherapy, Anal cancer, Toxicities, SIB, Sequential boost, Fecal incontinence

## Abstract

**Purpose:**

To compare toxicity of radiotherapy (RT) with concomitant chemotherapy (CHT) in patients (pts) with anal cancer treated with simultaneous integrated boost (SIB) versus sequential boost (SeqB).

**Methods:**

Sixty-six patients were treated from 2007 to 2021. Thirty patients underwent to SeqB concurrent to CHT and 37 to SIB-group. Toxicity assessment has been considered in acute and in late toxicities for gastrointestinal (GI), genitourinary (GU), cutaneous (CU) districts, according to Common Terminology Criteria for Adverse Events (CTCAE), Version 5.0. The Wexner scale among summary scoring systems has been used as a tool to measure fecal incontinence. The chi-square test for ordinal variables were used to evaluate the association between patient and treatment characteristics and acute or late severe toxicity. Univariable logistic regression models were fit to evaluate predictive factors associated with fecal incontinence.

**Results:**

Median follow-up was 61.5 months (IQR, 27.1–121.7 months) for all patients. Severe acute toxicity (≥ G2) was observed in 49 patients (74.2%). Late toxicity (≥ G2) occurred in 13 cases (19.6%). In assessment of cutaneous toxicity, there was also a significant reduction in ≥ G1 in SIB group with 29 patients (80.5%) vs SeqB group with 29 patients (96.6%) (p-value = 0.046). Of both groups 11 patients (16.6%) developed fecal incontinence, 8 (22%) in the SIB group and 3 (10%) in the SeqB.

**Conclusion:**

SIB for anal cancer treatment results in reduced acute and late cutaneous toxicity compared to SeqB. According to our results the rate of other acute and late toxicities are low and comparable between the two groups.

## Introduction

Anal cancer is a malignancy with a good prognosis, constituting 5% of all lower gastrointestinal tract malignancies. Despite comprising only 1.5% of all digestive system malignancies, the incidence has been increasing both in the USA and globally and is predominant in women [1, 2]. Historically, abdominoperineal resection (APR) with permanent colostomy has been the standard treatment [3]. Therapy requires a multidisciplinary approach, but the current standard of care is definitive chemoradiation, with APR reserved only as salvage treatment in persistent or locally recurrent disease [4]. Treatment of non-metastatic anal cancer has evolved since Nigro et al. reported complete responses after radio-chemotherapy, with a high rate of local control and the additional advantage of preserving a functioning anal sphincter [5]. Three randomized trials established radiotherapy and a concurrent chemotherapy regimen with 5-fluoruracil (5-FU) and mitomycin C (MMC) as the gold standard for treating anal carcinoma; however, these studies also reported significant acute toxicity rates. Patients treated with curative radiochemotherapy presented locoregional failure rates of approximately 30%, with disease-free survival (OS) rates of 60–75% [6–8]. Furthermore, advancements in radiation treatment over time have attempted to better optimize its delivery to potentially reduce toxicity and allow for maximal dose escalation. Sequential boost (SeqB) and simultaneous integrated boost (SIB) are the two most common radiotherapy techniques used to deliver different doses of radiation to different parts of the target area. SeqB deliver a lower dose of radiation to the entire target area first, followed by a higher dose of radiation delivered only the specific tumor area. SIB, conversely, delivers the entire course of treatment, including a higher dose of radiation to the tumor (boost), in a single session. To date, scientific literature currently offers limited comparative studies between those two radiation therapy techniques. The purpose of this retrospective analysis in a single-center study is to present a comparison of patients treated with sequential boost (SeqB) versus simultaneous integrated boost (SIB). This comparison will be made in terms of the assessment of acute and early-late toxicity and chronic sequelae, with particular care given to skin toxicity and fecal incontinence, after radiation therapy with concurrent chemotherapy.

## Methods

We retrospectively reviewed 66 patients with cancer of the anal canal or anal verge and good performance status (PS 0–1) treated at our institution between August 2007 and July 2021. Patient clinical data were obtained retrospectively from electronic and written records. Pre-treatment evaluation included history, physical examination, whole body computed tomography (CT), colonoscopy, proctoscopy examination, endorectal ultrasound (US), and magnetic resonance imaging (MRI) of the pelvis. Tumor histology was proven by biopsy. Patients were staged according to the 2002 American Joint Commission on Cancer (AJCC) staging system for anal cancer. Squamous cell carcinoma and basaloid carcinoma were included. Human immunodeficiency virus (HIV), human papillomavirus (HPV) viral status, or a history of acquired immune deficiency syndrome (AIDS) and other comorbidities were recorded to supplement staging information. Patients with distant metastases (M1) were excluded. We analyzed two cohorts of patients treated either with “shrinking field technique”: SeqB or SIB. The first cohort included 30 patients treated with SeqB and the second cohort included 36 patients treated with SIB. All cases were discussed at multidisciplinary meetings in the presence of medical oncologists. All patients gave written and informed consent before starting treatment. Acute toxicities during treatment were collected from a weekly on-treatment and radiation completion diary, while late toxicities were collected post-treatment from radiation and medical oncology follow-up documentation. Follow-up was regularly performed by radiation oncologists at 3 months after the end of radiotherapy and then every 4 months for the first 2 years and every 6 months up to 5 years, according to clinical conditions. Acute toxicity was defined as a treatment effect occurring between the start of radiation therapy and 12 weeks after treatment completion. Late toxicities were defined as occurring from 12 weeks through 2 years after treatment. The acute toxicities evaluated were localized on the skin in the gastrointestinal (GI), genitourinary (GU), and hematological (HT) districts. Late-developing effects included the same districts with the addition of subcutaneous toxicity and an assessment of fecal incontinence. The Common Terminology Criteria for Adverse Events v5.0 (CTCAE v5) quantified toxicity [9]. According to the severity of symptoms, fecal incontinence can be classified as major or minor incontinence. Major incontinence is the involuntary loss of stool. Minor incontinence consists of the loss of control of gas or occasional liquid stool soiling. The Wexner scale, among summary scoring systems, has been used as a tool to measure fecal incontinence [10].

### Radiotherapy

Treatments were designed using Eclipse software (Varian Medical Systems). Treatment plans were reviewed to identify each patient’s total radiation dose and doses to primary and boost planning target volumes (PTV). Radiation treatment plans were individually tailored for each patient. Two clinical target volumes (CTVs) were defined on the planning CT images. The gross tumor volume (GTV) was contoured on the CT scan and co-registered with MRI images and 18FDG PET-CT when available. Clinical target volume high-risk (CTV-HR) included the GTV plus a margin of 10–15 mm to include the entire anal canal, the peri-anal region, and the meso-rectum. If present, positive lymph nodes were included in the CTV-HR. CTV_pelvic was contoured by an expansion of 10–15 mm around the bilateral inguinal, femoral, external iliac, internal iliac, and common iliac vessels. PTV_pelvic and planning target volume high-risk (PTV-HR) were contoured by adding an isotropic expansion of 10 mm to the CTVs. CTV and PTV were contoured according to Radiation Therapy Oncology Group (RTOG) and Australasian Gastrointestinal Trials Group (AGITG) guidelines [11, 12].

Because three-dimensional conformal radiation therapy (3 DCRT) was the most commonly used treatment modality in the 2000s, the first cohort of patients primarily received 3 DCRT. The second cohort of patients received mostly Intensity-modulated radiation therapy (IMRT) treatment. The 3DCRT treatments were done with CT imaging data to better identify the intended target. All patients who received IMRT or VMAT received image-guided RT (IGRT).

#### SEQ

Thirty patients underwent SeqB. The sequential treatment consists of a first dose delivered in 25 fractions on PTV_pelvi and PTV_HR prescribed to 45 Gy (1.8 Gy daily fractions), followed by an additional dose delivered in 8 fractions on PTV_HR prescribed to 14.4 Gy in 1.8 Gy daily fractions (boost dose, PTV_boost). Total duration of treatment: 33 fractions. There was no planned break between radiation therapy to the entire pelvis and the EBRT boost.

#### SIB

The SIB-group included 36 patients from the most recent patient cohort (those treated since June 2012). The dose delivered to PTV_pelvic was 45 Gy (1.8 Gy daily fractions) with a simultaneous integrated boost to PTV_HR of 55 Gy (2.2 Gy daily fractions) in 25 fractions.

### Chemotherapy

All patients received concurrent chemoradiotherapy (CH-RT). The indication and choice of chemotherapy regimen were left to the referring medical oncologist. Based on practice variability, two types of regimens were administered as follows: capecitabine and bolus mitomycin C (MMC) or 5-fluorouracile (5-FU) and bolus MMC. In detail, based on performance and comorbidities, it consisted of: capecitabine 1650 mg/m2 orally as a 5-day/week regimen concomitant with RT; mitomycin 10 mg/m2 days 1 and 29; and 5-fluorouracil 1000 mg/m2 per day as an i.v. continuous infusion days 1–4 and 29–32.

## Statistical analysis

The chi-square test (or Fisher’s exact test, when appropriate) for ordinal variables was used to evaluate the association between patient and treatment characteristics and acute or early late severe toxicity (i.e., ≥ G2). Logistic regression was used for continuous variables in the comparison between acute and late toxicity and radiotherapy treatments (SIB vs sequential boost). For patients in the study, univariable logistic regression models were fit to separately evaluate individual predictive factors associated with fecal incontinence. Associations were summarized by calculating odds ratios (ORs) and corresponding 95% confidence intervals (CIs) from the model parameter estimates. Only significant variables from the univariate analysis were included in the multivariate analysis. P values of 0.05 or less were considered statistically significant. Statistical analysis was performed using the SPSS statistical software package, version 25.0 (SPSS, Chicago, IL, USA). Local control (LC), and colostomy-free survival (CFS) was calculated from the date of the end of radiotherapy course to the date of locoregional recurrence, salvage surgery or the date of the last follow-up. The Kaplan–Meier method was used to estimate the rates survival analysis.

## Results

### Characteristics of patients and treatments

Sixty-six patients with anal cancer were treated with curative radiotherapy. Table 1 summarizes the demographic and clinical characteristics of the patients. Thirty patients (45.4%) were treated with SeqB (years 2007–2012) and 36 patients (54.5%) with SIB (years 2012–2021). Thirty-two (48.5%) patients were treated with 3D-CRT, 32 (48.5%) patients with IMRT, and two (3%) patients with VMAT. Patients from both treatment groups were of similar age, with a similar distribution of gender, T stage, and TNM stage. The median dose for the elective low-risk PTV, which included bilateral external iliac, internal iliac, presacral, and inguinal nodes, was 45 Gy; the median dose for the high-risk PTV, which included strictly adjacent tumor tissues volume, was 59.4 Gy (IQR 55–59.4 Gy), with tumor and positive nodes receiving a boost dose up to a total median dose of 59.4 Gy (IQR 55–59.4 Gy). Twenty-six patients (39.3%) were treated with a combination of 5-FU and bolus MMC according to the Nigro protocol, and 40 patients (60.6%) were treated with a combination of capecitabine and bolus MMC. Furthermore, FU-5 was used in more patients with SeqB (sequential boost: 63.3% vs. 19.4% with SIB, p = 0.001). Eleven patients (16.6%) required a treatment break during radiation therapy (10 patients in SeqB group and only one patient in SIB group), which lasted a median of 5 days (IQR, 7–10 days). However, all patients in the study completed treatment. Salvage surgery was performed on nine patients (13.6%). Three of these are for toxicity, the remainder for disease recurrence. The median time from the end of RT to surgery was 9.23 months (IQR: 4.2–10.2).

**Table 1:** Clinicopathological characteristics of the patients

Characteristics of 66 patients	N (%) median (IQR)
Follow-up (median; months)	61.5 (27.1–121.7)
Age (median) < 60 year-old > 60 year-old	61 (50–69) 29 (43.9) 37 (56.1)
Gender	
Male Female	17 (25.8) 49 (74.2)
HPV	
Positive Negative	21 (31.8) 45 (68.2)
Stage	
I II III IV	6 (9.1) 40 (60.6) 12 (18.2) 8 (12.1)
Primary histology	
Squamous Basaloid Other	51 (77.3) 7 (10.6) 8 (12.1)
Technique	
3CRT IMRT VMAT	32 (48.5) 32 (48.5) 2 (3)
RT	
SIB Sequential boost	36 (54.5) 30 (45.4)
RT treatment duration (days)	
SIB Sequential boost	35.5 (35–36.7) 50 (47.5–55.5)
Total RT dose (Gy)	59.4 (55–59.4)
PTV (cc)	
High risk Low risk	229.9 (159.2–318.6) 1809.5 (1469.8–2291)
Systemic therapy	
5FU-MMC MMC- capecitabine	26 (39.3) 40 (60.6)

*HPV* Human Papilloma Virus, *ECOG-PS* Eastern Cooperative Oncology Group performance status, *RT* radiotherapy, *3DCRT* three-dimensional conformal radiation therapy, *IMRT* Intensity modulated radiation therapy, *VMAT* Volumetric modulated arc therapy, *SIB* simultaneous integrated boost, *PTV* planning target volume, *5FU* 5-fluorouracil, *MMC* Mitomycin C

## Toxicity

Relevant side effects were reported by the majority of patients. Severe acute toxicity (≥ G2) was observed in 49 patients (74.2%). Late toxicity (≥ G2) occurred in 13 cases (19.6%). Age at diagnosis and systemic therapy were statistically significant with a p-value of 0.049 and 0.033, respectively. Patients over 60 years receiving 5FU-MMC developed ≥ G2 acute toxicity. There were no treatment-related deaths. Radiation dose and PTV in cc did not show a significant factor. Tables 2 and 3 show the relationships between acute and late toxicities and RT treatments. Continuous variables analyzed with logistic regression did not show any statistical significance between the two types of radiotherapy treatments in terms of acute toxicity. In assessment of cutaneous toxicity, there was also a significant reduction in ≥ G1 in the SIB group with 29 patients (80.5%) compared with the SeqB group with 29 patients (96.6%) (p-value = 0.046). Instead, in the analyses of late toxicities, SeqB resulted in significantly fewer low-grade gastrointestinal toxicities compared to SIB with a p-value of 0.032 (OR 0.41; 95%CI: 0.18–0.92). Twenty-one patients (58.3%) showed ≥ G1 in the SIB group vs 10 patients (30.3%) in the SeqB (p-value = 0.043). Skin and hematological toxicity > G1 were higher in the SeqB with p values of 0.001 and 0.011, respectively. The majority of this toxicity was hematologic and cutaneous in nature and closely associated with patients receiving 5-FU as systemic therapy. In both groups, the total of grade 3 toxicity was moderate: 11 (30.5%) in the SIB group and 10 (33.3%) in the sequential boost group. Only two patients developed gastrointestinal G4 toxicity. Patients were given a treatment break because of perianal and anal discomfort and gastrointestinal-related symptoms (diarrhea, nausea, vomiting, and dehydration).

**Table 2:** Relationships between acute toxicity and RT treatments

Acute Toxicities	SIB N of pts 36 (54.5%)	Sequential boost N of pts 30 (45.5%)	P-value (OR; CI95%)
Genito-urinary (continuous)			0.626 (1.16;0.62–2.16)
≥ G1 ≥ G2 ≥ G3	22(61.1%) 8(22.2%) 0	18(60%) 9(30%) 0	0.927 0.472 0
Gastro-intestinal (continuous)			0.808 (0.93; 0.51–1.66)
≥ G1 ≥ G2 ≥ G3	31(86.1%) 16(44.4%) 2(5.5%)	27(90%) 13(43.3%) 1(3.3%)	0.632 0.928 0.666
Cutaneus (continuous)			0.086 (1.63; 0.93–2.87)
≥ G1 ≥ G2 ≥ G3	29 (80.5%) 19(52.7%) 5 (13.8%)	29(96.6%) 20(66.6%) 7(23.3%)	**0.046** 0.253 0.322
Haematological (continuous)			0.362 (1.32; 0.73–2.3)
≥ G1 ≥ G2 ≥ G3	10(27.7%) 6(16.6%) 1(2.7%)	14(46.6%) 4(13.3%) 2(6.6%)	0.112 0.707 0.45

Bold fonts significant values

*SIB* simultaneous integrated boost, *N* number, *OR* odds ratio

**Table 3:** Relationships between late toxicity and RT treatments

Late Toxicities	SIB N of pts 36 (54.4%)	Sequential boost N of pts 30 (45.5%)	P-value (OR; CI95%)
Genito-urinary (continuous)			0.216(0.52;0.19–1.45)
≥ G1 ≥ G2 ≥ G3	10(27.7%) 2(5.5%) 0	4(13.3%) 1(3.3%) 0	0.153 0.666 0
Gastro-intestinal (continuous)			**0.032 (0.41;0.18–0.92)**
≥ G1 ≥ G2 ≥ G3	21(58.3%) 5(13.8%) 2(5.5%)	10(30.3%) 1(3.3%) 0	**0.043** 0.137 0.190
Cutaneus (continuous)			**0.004 (4.40;1.6–12)**
≥ G1 ≥ G2 ≥ G3	4(11.1%) 1(2.7%) 1(2.7%)	17(56.6%) 4(13.3%) 0(0%)	** < 0.001** 0.107 0.358
Haematological (continuous)			0.073(3.66;0.8–15)
≥ G1 ≥ G2 ≥ G3	1(2.7%) 1(2.7%) 0	7(23.3%) 2(6.6%) 0	**0.011** 0.45 0
Fecal Incontinence			
Yes	8(22%)	3(10%)	0.185

Bold fonts significant values

*SIB* simultaneous intagreted boost, *N* number, *OR* odds ratio

## Fecal incontinence

Eleven patients (16.6%) developed fecal incontinence: 8 (22%) in the SIB group and 3 (10%) in the SeqB group (p-value 0.185). The univariate analysis results of predictive factors for fecal incontinence in all patients are included in Table 4. Among the clinical characteristics investigated, none were statistically significant. RT duration (< 37 vs ≥ 37 days) had a borderline effect on correlated fecal incontinence (p-value: 0.058; OR 0.25; 95%CI: 0.06–1.04). The continuous variable of RT dose had the same p-value of 0.054 (OR 0.99, 95%CI 0.99–1). Because there were no significant intervariable associations found on univariate analysis, a multivariate analysis was not performed.

**Table 4:** Univariate analysis of predictive factors associated with fecal incontinence

Variable	Classification	N patients, (%)	p-value	OR	95% CI
Age	> 60 < 60	6 (16.2) 5 (5.8)	0.912	0.92	0.25–3.40
Gender	Male Female	1 (5.8) 10(20.4)	0.195	4.1	0.48–34.7
ECOG-PS	< 1 ≥ 1	10 (21.2) 1 (5.8)	0.146	0.20	0.02–1.7
Stage	I-II III-IV	9 (19.5) 2 (10)	0.347	0.45	0.08–2.32
Site	Anal canal anal verge	6 (16.2) 5 (5.8)	0.406	0.40	0.04–3.4
RT duration (days)	< 37 ≥ 37	8 (26.6) 3 (8.3)	0.058	0.25	0.06–1.04
Technique	3DCRT IMRT VMAT	3 (9.3) 8 (25) 0	0.085	0.29	0.07–1.8
PTV HR (cc)	Continuous		0.07	0.99	0.98–1
	< 230 ≥ 230	8 (23.5) 3 (9.3)	0.135	0.33	0.08–1.4
PTV LR (cc)	Continuous		0.292	0.99	0.98–1.1
	< 1800 ≥ 1800	6 (18.7) 5 (14.7)	0.66	0.74	0.20–2.7
Dose RT (Gy)	Continuous		0.054	0.99	0.99–1
	< 59.4 ≥ 59.4	8 (25.8) 3 (8.5)	0.073	0.27	0.06–1.2
Salvge Surgery	Yes No	1 (11.1) 10 (17.5)	0.634	0.58	0.06–5.2

*OR* odds ratio, *CI* confidence interval, *ECOG-PS* Eastern Cooperative Oncology Group performance status, *RT* radiotherapy, *3DCRT* three-dimensional conformal radiation therapy, *IMRT* Intensity modulated radiation therapy, *VMAT* Volumetric modulated arc therapy, *PTV* planning target volume

## Survival outcomes

**Fig. 1:**
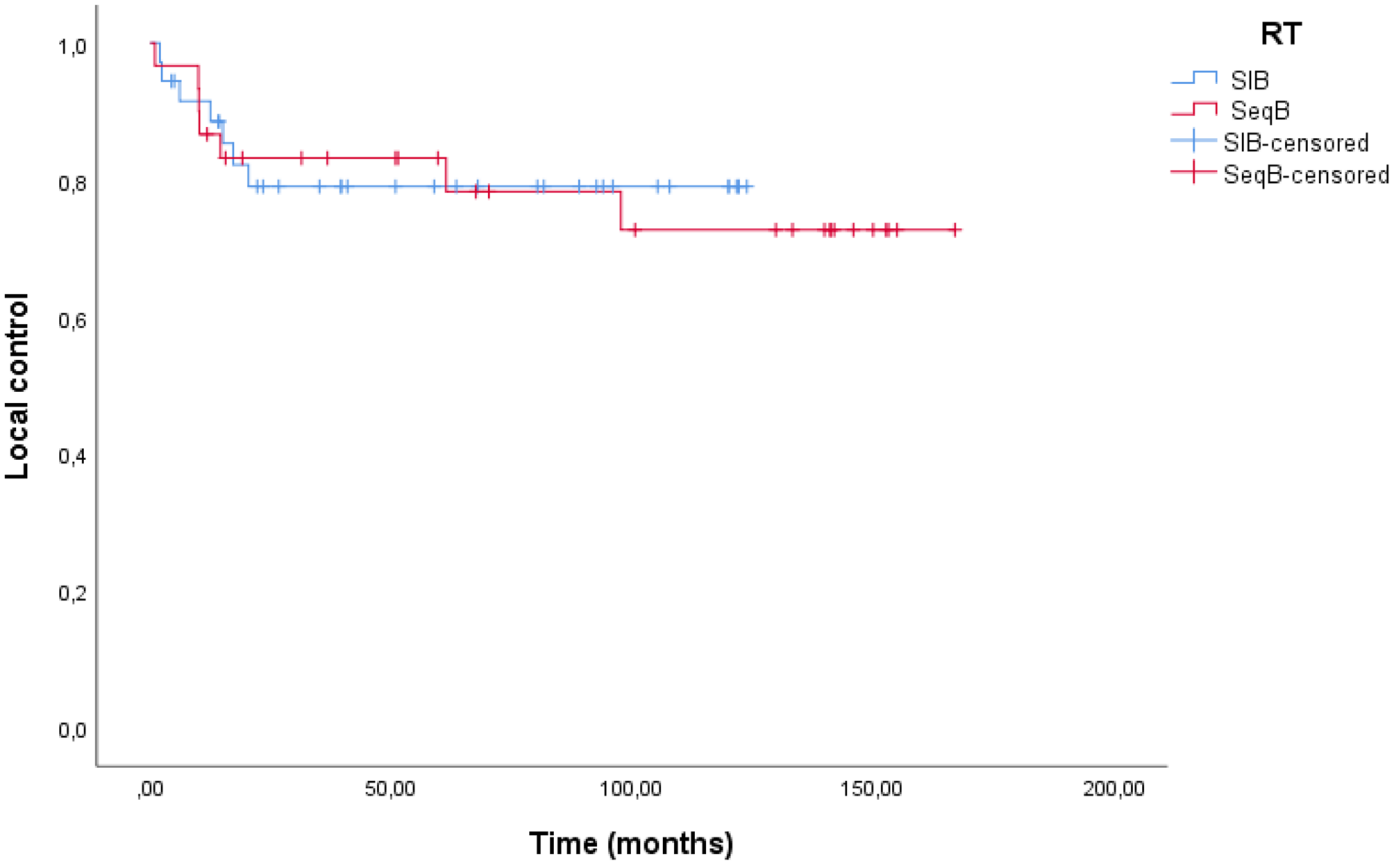
Kaplan–Meier curves of Local Control of patients in the SIB group vs SeqB group

**Fig. 2:**
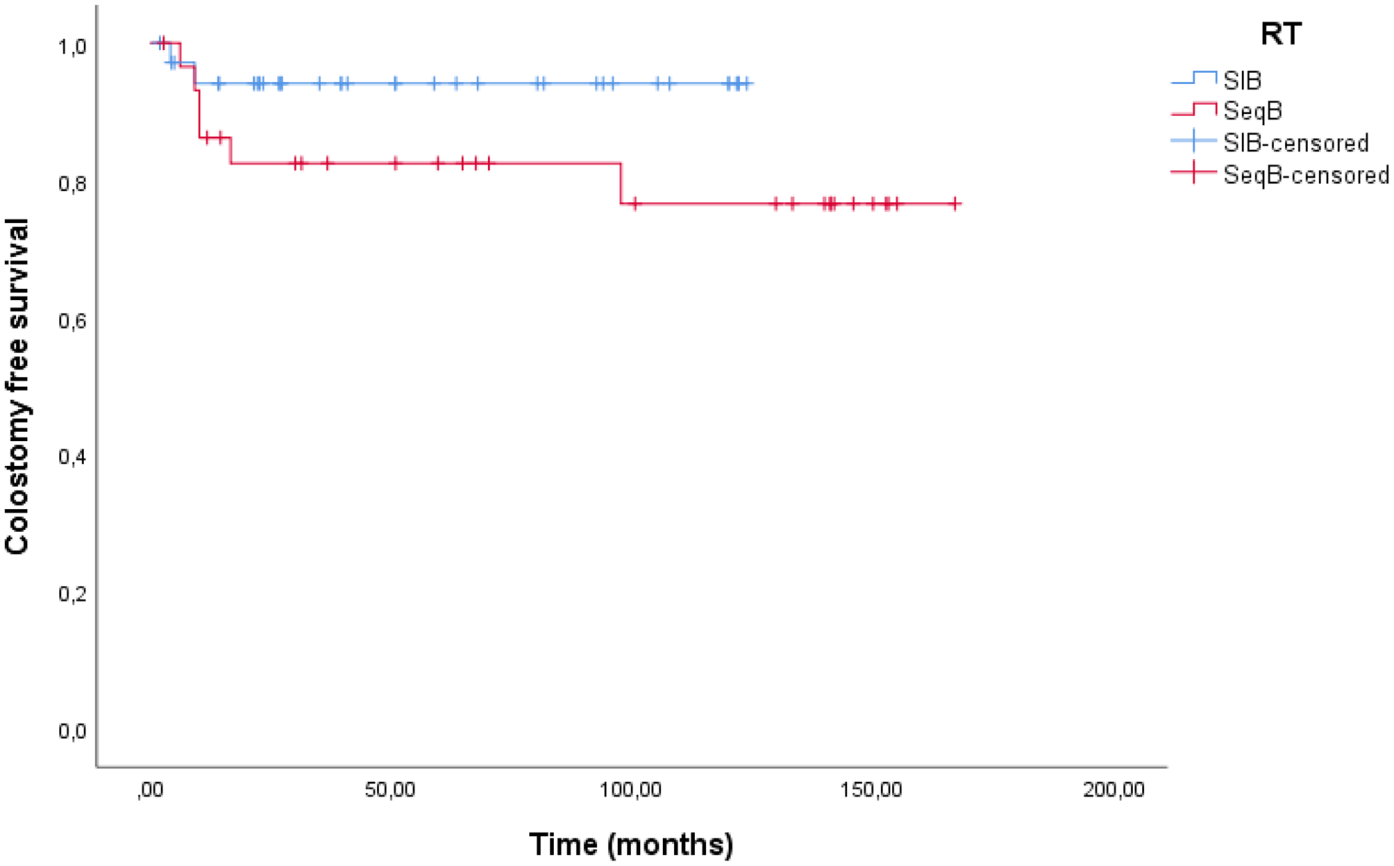
Kaplan–Meier curves of Colostomy free survival of patients in the SIB group vs SeqB group

The median follow-up was 61.5 months (IQR, 27.1–121.7 months) for all patients. Median LC was not reached, and the, 1 year, 2 year and 5 years were 89.2%, 80.9%, and 78.4%, respectively. Median CSF was not reached and the 6 months, 1 year, and 2 year were 98.4%, 90.4%, and 88.6%, respectively. The 6 months and 1-year LC for SIB group vs. SeqB group were 88.5%, and 79.1%, vs. 83.2%, and 78.3%, respectively (p = 0.873) (Fig. 1). The 6 months and 1-year CSF for SIB group vs. SeqB group were 97.1%, and 94.1%, vs. 86.2%, and 82.5%, respectively (p = 0.128) (Fig. 2).

## Discussion

Concurrent chemoradiation represents the standard treatment for localized anal cancer, providing local control and survival rates similar to those reported with radical surgery. SeqB and SIB are the two most frequently used radiotherapy fractionation protocols in daily clinical practice for treating pelvic malignancies and delivering different levels of doses to cover different parts of the targets. Both methods are effective treatment strategies in the combined modality therapy of anal cancer patients [13]. To the best of our knowledge, there is no direct prospective randomized controlled comparison and only few heterogeneous retrospective data investigating SIB vs. sequential boost in anal cancer. Despite the increasing widespread of IMRT and VMAT radiation techniques allowing SIB treatments, SeqB delivered using 3D-CRT fields is yet commonly used especially in peripheral-spoke hospitals or in middle/low-income countries radiation departments. Thus, the importance of our series comparing those two strategies.

In the present study, 66 consecutive patients were evaluated after treatment with SIB and SeqB. Severe acute toxicity was observed in 49 patients (74.2%). Late severe toxicity occurred in 13 cases (19.6%). In the assessment of cutaneous acute toxicity, there was a significant reduction in ≥ G1 in the SIB group with 29 patients (80.5%) compared with the sequential boost group with 29 patients (96.6%) (p-value = 0.046). Taking in consideration that most of SeqB in our cohort were treated with 3DCRT, this data is in line with RTOG 9811 using 3DCRT and reporting the cutaneous toxicity as the most common toxicity [14]. Similarly, to our SIB cohort, in RTOG 0529 using IMRT severe skin toxicities were significantly reduced [15]. Another possible explanation of the higher skin toxicities rates could be the prevalence of 5-FU as systemic therapy in SeqB cohort which is shown to be significantly related to increased skin toxicities in other mentioned studies.

In our study 86.1% of patients in the SIB group and 90% in the SeqB group had acute GI toxity. Instead, as far as late GI toxicity is concerned, 58.3% was found in the SIB group and 30.3% in the SeqB group. Only 2 (5.5%) patients in the SIB group showed late G toxicity > G3resolved with drug therapy. Literature reports show late toxicity rates ranging from 4 to 50%, depending on follow-up length, radiotherapy technique, dose per fraction, and combination of chemotherapeutic agents [16]. In the Italian study by Dell’Acqua et al., data on early-late toxicity were collected for 73 subjects (87%). Severe GI toxicity (≥ G3), including diarrhea, was observed in two patients (3%) and severe vulvo-vaginal toxicity was observed in only two (3%) patients [17]. In our study, late toxicities, gastrointestinal and cutaneous, were statistically significant with a p-value of 0.043 and < 0.001, respectively. One possible explanation could be a different distribution of radiation to the bowel receiving higher doses when using IMRT-SIB compared to 3DCRT. Moreover, IMRT-SIB leads higher volumes to bowel to receive low doses of radiation which may increase the probability of bowel infiammation.

The incidence of the side effects found in the current study was comparable with that observed in other series. Call et al. reported an acute severe GI toxicity of 12%, and Salama et al. reported a severe skin toxicity of 37.7% [18, 19].

In our analysis treatment time was markedly shorter in the SIB group vs SeqB group (median 35.5vs50 days). The number of treatment discontinuations was also higher in the SeqB group with a median duration of break of 5 days. However all patients complete the treatment course without a significant delay in both groups.

Our study showed an excellent LC. Globally we reported a 1 year, 2 year and 5 years of LC of 89.2%, 80.9%, and 78.4%, respectively and 6 months, 1 year, and 2 year of CSF of 98.4%, 90.4%, and 88.6%, respectively. Our analysis did not show any statistically significant different in LC e CSF between SIB and SeqB cohorts. The 6 months and 1-year LC for SIB group vs. SeqB group were 88.5%, and 79.1%, vs. 83.2%, and 78.3%, respectively. The 6 months and 1-year CSF for SIB group vs. SeqB group were 97.1%, and 94.1%, vs. 86.2%, and 82.5%, respectively. Our results stand in line with a literature that using radiotherapy and concurrent chemotherapy [20, 21].

Fecal incontinence is a well-known late effect after pelvic radiotherapy, but prevalence varies widely, with rates ranging from 3 to 53% [22]. In the study of Bentzen et al., incontinence for gas was significantly more common among survivors than volunteers (55% vs. 12%). Incontinence for liquid stools occurred in 41% vs. 5%, and incontinence for solid stools occurred in 15% of survivors but none of the volunteers [23]. In our study, 11 patients (16.6%) developed fecal incontinence. In the univariate analysis of predictive factors, RT duration and RT dose had a borderline effect on correlated fecal incontinence with a p-value of 0.058 and 0.054, respectively. In our series we found a different between SIB and SeqB when analyzing fecal incontinence, 22% vs 10% respectively although not reaching statistical significance. Supporting this hypothesis, at univariate analysis, shorter duration of treatment and higher doses were borderline related to had a higher rate of facal incontinence.

Our study has some limitations represented mainly by the retrospective nature of the analysis, the small sample of patients included and the different distribution among the two cohorts of 3DCRT and IMRT as a potential selection bias.

## Conclusion

In the present study, SIB for anal cancer treatment results in reduced acute and late cutaneous toxicity compared to SeqB. According to our results, the rates of other acute and late toxicities are low and comparable between the two groups. There is a lack of data in the literature on the comparative toxicity of both treatments, which should be investigated in future clinical trials with longer follow-up periods.

## Data Availability

Not applicable here.
